# The radiation environment of anaesthesiologists in the endoscopic retrograde cholangiopancreatography room

**DOI:** 10.1038/s41598-019-45610-4

**Published:** 2019-06-24

**Authors:** Bora Lee, Min-Soo Kim, Darhae Eum, Kyeong Tae Min

**Affiliations:** Department of Anaesthesiology and Pain Medicine, Severance Hospital, Anaesthesia and Pain Research Institute, Yonsei University College of Medicine, 50-1 Yonsei-ro, Seodaemun-Gu, Seoul, 03722 Republic of Korea

**Keywords:** Risk factors, Occupational health

## Abstract

Anaesthesiologists are increasingly involved in nonoperating room anaesthesia (NORA) for fluoroscopic procedures. However, the radiation exposure of medical staff differs among NORA settings. Therefore, we aimed to investigate the radiation environment generated by fluoroscopic endoscopic retrograde cholangiopancreatography (ERCP) and the radiation exposure of anaesthesiologists. The dose area product (DAP), radiation entrance dose (RED), and fluoroscopy time (FT) according to the procedures and monthly cumulative radiation exposure were analysed at two sites (neck and wrist) from 363 procedures in 316 patients performed within 3 months. The total RED and DAP were 43643.1 mGy and 13681.1 Gy cm^2^, respectively. DAP and RED (r = 0.924) were strongly correlated and DAP and FT (r = 0.701) and RED and FT (r = 0.749) were moderately correlated. The radiation environment per procedure varied widely, DAP and RED per FT were the highest during stent insertion with esophagogastroduodenoscopy. Monthly cumulative deep dose equivalents at the wrist and neck ranged between 0.31–1.27 mSv and 0.33–0.59 mSv, respectively, but they were related to jaw thrust manipulation (r = 0.997, *P* = 0.047) and not to the radiation environment. The anaesthesiologists may be exposed to high dose of radiation in the ERCP room, which depends on the volume of procedures performed and perhaps the anaesthesiologists’ practice patterns.

## Introduction

An increasing number of anaesthesiologists are performing sedation during fluoroscopic procedures under nonoperating room anaesthesia (NORA) compared with that in the past^1^. Among these procedures involving NORA, there is a growing number of radiation-assisted procedures, especially those performed in the endoscopic retrograde cholangiopancreatography (ERCP) room^2,3^. A previous study reported that the radiation environment differed according to the NORA setting; it was higher for the cardiac catheterisation laboratory than for interventional radiology and higher for monitored sedation than for the general anaesthesia setting^4^.

Therefore, we aimed to investigate the radiation environment generated by fluoroscopic ERCP procedures and the radiation exposure of anaesthesiologists who are involved in monitored anaesthesia care during their everyday practice.

## Methods

### Ethics approval

This retrospective study conducted at a tertiary university hospital was approved by the Severance Hospital institutional review board (protocol number: 4–2017–0920). The need to obtain written consent from subjects was waived.

### Study population

Three anaesthesiologists were involved in monitored anaesthesia care for 363 procedures in 316 patients performed by 10 endoscopists in the ERCP room over 3 months, between July and September 2017. The procedures took place from 9 AM to 4 PM in monthly shifts. All three anaesthesiologist who were involved in monitored anaesthesia care were senior residents with at least 3 years of anaesthesia experience, and they were supervised by specialist anaesthesiologists.

### Conduct of the study

Various procedures were performed under fluoroscopy, such as luminal dilation, stent insertion with esophagogastroduodenoscopy, diagnosis, bile duct dilation and stent, stone removal with endoscopic retrograde cholangiopancreatography, and percutaneous transhepatic choledochoscopic stone removal. The patients were sedated using a standardised protocol of continuous propofol (Fresofol 1% MCT injection, Fresenius Kabi Korea, Seoul, South Korea) infusion with intermittent administration of fentanyl (fentanyl citrate, Hana Pharm Co., Ltd., Seoul, South Korea). Electrocardiography, pulse oximetry (blood oxygen saturation [Spo_2_]), non-invasive arterial blood pressure, and capnometry using a nasal prong were employed as standard monitoring methods. Oxygen was administered through a nasal prong, with a flow rate of 5 L/min. If oxygen saturation decreased (Spo_2_ < 90%), the anaesthesiologist reduced the rate of propofol infusion and performed jaw thrust until the patient breathed well. If oxygen saturation was not restored (Spo_2_ > 90%) after jaw thrust, a nasopharyngeal airway was inserted.

The anaesthesiologist wore a lead apron and thyroid shield for personal protection. The usual distance between the wrist of the anaesthesiologist and the central beam of the X-ray system ranged between 80 and 120 cm unless airway manipulation was required. When airway manipulation was required, the distance was reduced to between 40 and 60 cm. Thermoluminescent dosimeters (UD-802, Panasonic Communications Kyushu Co., Ltd., Ueda Usa, Oita, Japan) were used to measure the cumulative radiation exposure of the anaesthesiologists at the neck outside the thyroid shield and the dominant wrist^4^. These thermoluminescent badges use the tissue equivalent (Li_2_B_4_O_7_:Cu) and are highly sensitive (CaSO_4_:Tm), making it possible to precisely measure various types of radiation over a wide range of doses (10 μSv–10 Sv). A dedicated reader (UD-716, Panasonic Communications Kyushu Co., Ltd.) obtains the measurements used to determine the deep dose equivalent, Hp(10) mSv^5^.

### Data collection and handling

During fluoroscopic procedures, radiation entrance dose (mGy), fluoroscopic time (s), and dose area product (Gy cm^2^) were automatically recorded by the fluoroscopy unit of the overcoach ceiling mounted system (Artis Zee Multi-Purpose, Siemens, München, Germany). The demographic data of patients, the types of procedures, and the radiation entrance dose, fluoroscopic time, and dose area product per procedure were retrospectively collected from electronic medical records.

### Statistical analysis

Data for the radiation environment are expressed as mean (standard deviation), median (interquartile range), or number of patients (%). Parametric data were analysed using one-way analysis of variance, whereas nonparametric data were analysed using the Kruskal-Wallis test. Categorical variables were evaluated using the chi-squared test or Fisher’s exact test, as appropriate. Correlation analysis between the radiation exposure of the anaesthesiologists (the deep dose equivalent) and radiation environment of the endoscopic retrograde cholangiopancreatography room or other variables was performed using the Pearson or Spearman methods. A P value < 0.05 was considered statistically significant. All analyses were performed using R version 3.4.1 (R Foundation for Statistical Computing, Vienna, Austria) and SPSS 23.0 (IBM, Armonk, NY, USA).

## Results

The total radiation entrance dose and dose area product of the radiation environment for the 3 months were 43643.1 mGy and 13681.1 Gy cm^2^, respectively. The fluoroscopy unit generated x-ray at 66–74 kV during the procedures. The most commonly performed procedure was bile duct dilation and stent insertion with endoscopic retrograde cholangiopancreatography, which accounted for 33% of all procedures. The radiation environments differed widely according to the type of procedure (Table 1). In detail, the median anaesthesia time (35 min), radiation entrance dose (107.6 mGy), fluoroscopic time (343 s), and dose area product (35.0 Gy cm^2^) were the highest for percutaneous transhepatic choledochoscopic stone removal. However, the radiation entrance dose per fluoroscopic time (0.59 mGy sec^−1^) and dose area product per fluoroscopic time (0.26 Gy cm^2^ sec^−1^) were the highest for stent insertion with esophagogastroduodenoscopy. In the correlation analysis among variables of the radiation environment, there was a strong correlation between the dose area product and radiation entrance dose (r = 0.924), but a weaker one between the dose area product and fluoroscopic time (r = 0.701) or the radiation entrance dose and fluoroscopic time (r = 0.749) (*P* < 0.001).

**Table 1:** Procedural radiation environments in the endoscopic retrograde cholangiopancreatography (ERCP) room.

Procedure	EGD-Dilation (N = 10)	EGD-Stent (N = 47)	ERCP-Diagnosis (N = 91)	ERCP-Dilation/Stent (N = 121)	ERCP-Stone removal (N = 59)	PTCS (N = 35)	P value
Anaesthesia time (min)	27.5 (15–35)	20 (15–25)	20 (15–30)	20 (15–30)	20 (18–28)	35 (25–40)	<0.001
Number of exposures	6 (5–9)	6 (4–9)	5 (3–9)	8 (5–11)	6 (4–7)	8 (6–11)	<0.001
FT (sec)	121 (5–283)	164 (79–266)	140 (31–299)	203 (83–349)	196 (107–275)	343 (184–510)	0.002
DAP (Gy cm^2^)	17.5 (1.6–30.1)	30.9 (17.8–70.6)	12.3 (3.4–31.7)	18.7 (9.7–45.9)	21.1 (11.8–35.3)	35.0 (23.6–69.9)	<0.001
RED (mGy)	43.3 (3.4–85.1)	84.0 (40.7–181.5)	47.2 (13.1–105.2)	64.1 (29.2–133.6)	64.3 (40.3–117.1)	107.6 (70.4–214.1)	<0.001
DAP per FT (Gy cm^2^ sec^−1^)	0.18 (0.13–0.32)	0.26 (0.11–0.43)	0.10 (0.08–0.16)	0.11 (0.08–0.17)	0.12 (0.08–0.15)	0.10 (0.09–0.19)	<0.001
RED per FT (mGy sec^−1^)	0.58 (0.35–0.62)	0.59 (0.32–0.99)	0.30 (0.25–0.52)	0.35 (0.25–0.52)	0.31 (0.25–0.52)	0.38 (0.25–0.62)	<0.001
Patient’s age (years)	61.0 (32.0–72.0)	63.0 (57.5–68.5)	64.0 (53.0–72.0)	62.0 (56.0–72.0)	64.0 (55.5–76.0)	66.0 (59.5–77.0)	0.329
Patient’s height (cm)	168.3 (9.8)	165.5 (7.3)	164.3 (8.8)	164.9 (9.3)	163.0 (9.8)	159.7 (8.5)	0.002
Patient’s weight (kg)	57.0 (12.4)	55.6 (11.0)	63.8 (12.0)	63.3 (11.6)	63.5 (12.0)	55.6 (11.0)	0.499

Values are presented as median (IQR) or mean (SD). FT, fluoroscopy time; DAP, dose area product; RED, radiation entrance dose; EGD, esophagogastroduodenoscopy; PTCS, percutaneous transhepatic choledochoscopic stone removal. N is the number of procedures. The P value denotes the probability that the quantity median for each procedure belongs to the same distribution.

**Figure 1:**
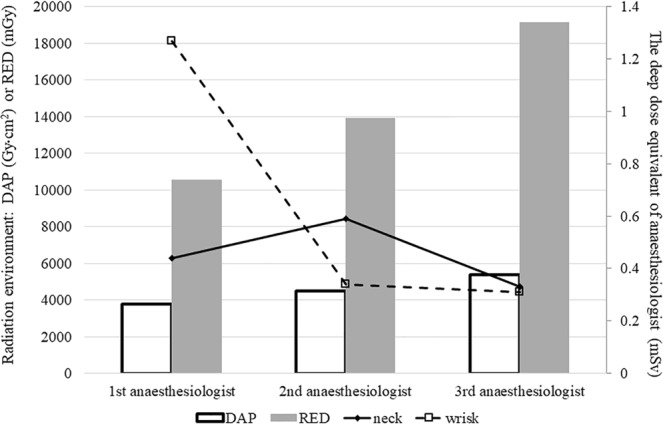
Total radiation entrance dose (RED) and dose area product (DAP) for the radiation exposure of each anaesthesiologist, and the effective radiation dose (neck and wrist) for each anaesthesiologist. The deep dose equivalents for the anaesthesiologists’ neck and wrist were not associated with the radiation environment variables such as total RED and DAP.

The age, height, and weight of the patients managed by the anaesthesiologists were similar across the 3 months. The number of patients with history of snoring and the fentanyl dose administered were highest in the first month. The first anaesthesiologist cared for the patients more often than the others by performing jaw thrust or nasopharyngeal airway insertion due to oxygen desaturation events (Table 2). Regarding the radiation environment for the anaesthesiologists, the cumulative dose area product, radiation entrance dose, and cumulative fluoroscopy time were in the order of the third, second, and first anaesthesiologist (Table 3). However, the personal dosimeter readings for the neck and wrist of the anaesthesiologists were not related to the cumulative radiation entrance dose and dose area product (Fig. 1). The highest radiation exposure of the neck was 0.59 mSv in the second anaesthesiologist and the highest radiation exposure of the wrist was 1.27 mSv in the first anaesthesiologist. The coefficient of correlation between radiation exposure of the wrists of the anaesthesiologists and jaw thrust manipulation was 0.997 (*P* = 0.047).

**Table 2:** Patient characteristics and procedural details per anaesthesiologist in the endoscopic retrograde cholangiopancreatography (ERCP) room.

	1st anaesthesiologist	2nd anaesthesiologist	3rd anaesthesiologist	P value
No. of procedure	109	118	136	
Age (year)	62 (16)	64 (13)	61 (14)	0.112
Female	37 (34)	34 (29)	49 (36)	0.463
Height (cm)	164.4 (9.7)	162.8 (12.0)	164.4 (9.3)	0.840
Weight (kg)	60.7 (12.5)	61.4 (11.6)	62.3 (12.1)	0.295
Body mass index (kg m^−2^)	22.4 (3.6)	22.8 (3.5)	23.0 (3.4)	0.193
ASA class >3	27 (25)	42 (36)	33 (24)	0.088
Emergency case	3 (2.8)	1 (1)	0 (0)	0.116
Snoring history	14 (13)	1 (1)	7 (5)	0.001
Jaw thrust due to oxygen desaturation event	14 (13)	6 (5)	5 (4)	0.012
Nasopharyngeal airway insertion	9 (8)	2 (2)	1 (1)	0.003
Anaesthesia time (min)	20 (15–25)	25 (20–30)	25 (15–30)	0.189
Propofol dose (mg kg^−1^)	2.4 (2.0–3.1)	2.5 (1.8–3.2)	2.6 (1.8–3.5)	0.591
Fentanyl dose (mcg kg^−1^)	1.6 (1.2–1.9)	0.9 (0.7–1.3)	1.4 (1.1–1.7)	<0.001

Values are presented as mean (SD), or patient number (%). The P value denotes the probability that the quantity for the different anaesthesiologists belongs to the same distribution.

**Table 3:** Total radiation exposure per anaesthesiologist in the endoscopic retrograde cholangiopancreatography (ERCP) room, for the various procedure types. FT, fluoroscopy time; DAP, dose area product; RED, radiation entrance dose; EGD, esophagogastroduodenoscopy; PTCS, percutaneous transhepatic choledochoscopic stone removal.

	1st anaesthesiologist				2nd anaesthesiologist				3rd anaesthesiologist			
	Number of procedures	FT (sec)	DAP (Gy cm^2^)	RED (mGy)	Number of procedures	FT (sec)	DAP (Gy cm^2^)	RED (mGy)	Number of procedures	FT (sec)	DAP (Gy cm^2^)	RED (mGy)
EGD-Dilation	5	581	110.4	232.3	0	—	—	—	5	1715	210.4	900.4
EGD-Stent	22	4061	1332.0	2789.8	20	5727	1155.9	2906.7	5	717	264.7	762.9
ERCP-Diagnosis	22	3094	319.9	960.3	26	6514	854.8	2646.0	43	13003	1438.7	5809.9
ERCP-Dilation/Stent	42	12133	1383.1	4574.5	43	10186	1524.0	5338.3	36	12303	1551.5	5344.5
ERCP-Stone removal	10	2666	309.6	963.5	17	4193	458.2	1484.3	32	7501	956.1	3355.2
PTCS	8	2525	334.7	1053.1	12	3967	508.8	1537.6	15	6632	968.3	2983.8
Total	109	25060	3789.7	10573.5	118	30587	4501.7	13912.9	136	41871	5389.7	19156.7

## Discussion

In this study, we investigated the radiation environment generated by fluoroscopic ERCP procedures and the radiation exposure of anaesthesiologists who were involved in monitored anaesthesia care setting.

For the radiation environment generated during fluoroscopy, dose area product, fluoroscopy time, and radiation entrance dose are commonly-used indicators of the radiation exposure of the patient. The radiation entering the patient, dose area product, is correlated with the radiation exposure of the medical staff and used as an indirect measure of the occupational dose they receive because the largest source of occupational radiation exposure for the medical staff is the scattered radiation from the patient^6–8^. However, it is unlikely that these parameters can be used for monitoring radiation exposure of anaesthesiologists working in the ERCP room. Although our results were obtained from a single institution, the radiation environment of each procedure differed significantly according to the types of procedures and the anaesthesiologists were working in environments with high risk of radiation exposure. None of the variables of the radiation environment fit the normal distribution curve, which implies that it is difficult to predict the radiation dose based only on the type of procedure. While percutaneous transhepatic choledochoscopic stone removal required the longest fluoroscopic time and produced the largest dose area product and radiation entrance dose, the dose area product and radiation entrance dose per fluoroscopic time were the largest during stent insertion with esophagogastroduodenoscopy. The total radiation dose of the radiation environment might be affected by multiple factors unmanageable by the anaesthesiologist, which has also been shown by other studies^2,9^. Nevertheless, there was a correlation between fluoroscopic time and the dose area product for endoscopic retrograde cholangiopancreatography procedures in our study, as also observed in other studies^2^. Although Ismail *et al*. revealed that the radiation exposure of anaesthesiologists in the endoscopic retrograde cholangiopancreatography suite was lower than that in the cardiac catheterisation laboratory, anaesthesiologists are still exposed to a considerable amount of radiation in the ERCP room^4^.

Regarding the radiation exposure of anaesthesiologists in the ERCP room, we made some interesting observations. First, the radiation exposure of the anaesthesiologists was not related to the radiation environment of procedures performed in the endoscopic retrograde cholangiopancreatography room. Although the dose area product and radiation entrance dose increased in the order of the first to the third anaesthesiologist, the deep dose equivalent at the neck and wrist were the highest in the second and first anaesthesiologist, respectively. This could imply that the radiation exposure of anaesthesiologists may not be influenced by the radiation environment of the ERCP procedure because we could not find any correlation between anaesthesiologists’ radiation exposure and the radiation entrance dose or dose area product. We obtained some indication as to the reason for this from the requirement for airway manipulation for the anaesthesiologist. The first anaesthesiologist performed jaw thrust and nasopharyngeal airway insertion more often than did the others. This was confirmed by the strong correlation (r = 0.997, *P* = 0.047) between radiation exposure and the wrists of the anaesthesiologists. Similarly, the total number of drug boli and infusion rate changes by the anaesthesiologist was significantly related to the radiation exposure of the anaesthesiologist in other study^10^. However, these events were not associated with radiation exposure of the neck. Then, we inquired as to why the radiation dose measured at the necks of the anaesthesiologists did not show a pattern similar to that for the incidence of airway manipulation. This could be explained by the fact that the radiation intensity is inversely proportional to the square of distance from the source of scattered radiation (in this case, the patient).

Second, the radiation exposure of anaesthesiologists during their everyday practice did not exceed the annual radiation dose recommended by the International Commission on Radiological Protection^11–13^. However, it is important for anaesthesiologists to be aware that their radiation exposure is related to the patients’ responses to sedation methods. It is worthwhile to refine the sedation protocol used during nonoperating room anaesthesia to avoid the respiratory depression of patients, especially in the ERCP room^14,15^. We also recommend that anaesthesiologists should stand as far as possible from the sources of scattered radiation.

Third, in our institution, the design and working of the ERCP room was not influenced by consideration of the anaesthesiologists’ perspective. The dosimeter at our institution is only for regular staff and not for anaesthesiologists, who work in monthly shifts. However, when anaesthesiologists are requested to participate in procedures related to fluoroscopy, they must monitor their radiation exposure and wear protective shielding because the monthly radiation exposure dose is not constant^10^. The lead apron covers almost all active bone marrow and major organs, such as the heart, lungs, and major vessels^3^. The thyroid shield protects the thyroid gland and the oesophagus, vertebrae, and bone marrow^12^. Anaesthesiologists should wear the radiation protection equipment, including the lead apron and thyroid collar, because it protects the most radiation-sensitive areas. The lens of the eye is vulnerable to radiation and cannot be covered by routine equipment^16–18^. Protection glasses are recommended especially when the anaesthesiologists stand near the patient, regardless of the number of ERCP procedures performed^10,12,19^.

Similar to our study, Ismail *et al*. also reported on the radiation exposure of anaesthesiologists during ERCP; the net radiation exposure of the neck was 0.25 mSv during 39 ERCP procedures (0.0064 mSv per procedure) performed over 6 months^4^ compared with 1.36 mSv during 363 procedures (0.0037 mSv per procedure) performed over 3 months in our study. This difference could be attributable to many factors influencing the radiation environment, such as the volume of the procedures as well as the anaesthesiologists’ practice pattern. We believe that there is a need for a guideline suggested by anaesthesiologists’ societies, such as the ones suggested by cardiologists’ societies^20^. For instance, considering that the radiation tolerance limit of pregnant women is 1 mSv after the pregnancy is declared, as suggested by the International Commission on Radiological Protection, the participation of a pregnant anaesthesiologist in the ERCP room should be limited^3,13^.

There are several limitations to our study. First, since this study was conducted at a single institute, our results may not be applicable to other institutes. Although the radiation environment related to the fluoroscopic procedure depends on many factors and the conditions differ across medical institutions, our data would be clinically meaningful considering that the radiation dose generated by each procedure was reviewed in a total of 363 cases. Second, we only measured the cumulative radiation exposure dose of anaesthesiologists over 3 months. Thus, it is difficult to identify factors affecting the radiation exposure of the anaesthesiologist. We believe that well-designed prospective studies monitoring the real-time radiation exposure of anaesthesiologists could identify the factors that influence the effective radiation dose of anaesthesiologists during fluoroscopic procedures^21^.

In conclusion, anaesthesiologists may be exposed to high risk of radiation exposure in ERCP room, which depends on the volume of performed procedures and perhaps the anaesthesiologists’ practice patterns. Therefore, the procedural parameters and the working environment of anaesthesiologists in fluoroscopic NORA settings must be considered to reduce radiation exposure, especially in high-volume centres, such as working in shifts, wearing protective equipment, dosimeter-based monitoring, and refining the sedation protocol. A future prospective study using real-time radiation dose monitoring is anticipated.

## Data Availability

The data that support the findings of this study are available from the corresponding author on reasonable request.
